# Laparoscopic versus Open Radical Cystectomy for Elderly Patients over 75-Year-Old: A Single Center Comparative Analysis

**DOI:** 10.1371/journal.pone.0098950

**Published:** 2014-06-05

**Authors:** Shuxiong Zeng, Zhensheng Zhang, Xiaowen Yu, Ruixiang Song, Rongchao Wei, Junjie Zhao, Linhui Wang, Jianguo Hou, Yinghao Sun, Chuanliang Xu

**Affiliations:** 1 From the Department of Urology, Changhai Hospital, Second Military Medical University, Shanghai, P. R. China; 2 From the Department of Geriatrics, Changhai Hospital, Second Military Medical University, Shanghai, P. R. China; University of British Columbia, Canada

## Abstract

**Purpose:**

To explore the morbidity, mortality and oncological results of laparoscopic radical cystectomy (LRC) in the elderly patients over 75-year-old in contrast with open radical cystectomy (ORC).

**Materials and Methods:**

We analyzed 46 radical cystectomies from January 2009 to December 2013 in patients over 75-year-old in our institute, 21 patients in the LRC group and 25 in the ORC group. Demographic parameters, operative variables and perioperative outcome were retrospectively collected and analyzed between the two groups. Perioperative morbidity and mortality were categorized as early (within 90 days after surgery) or late (more than 90 days) according to the time of occurrence.

**Results:**

Patients in both groups had comparable preoperative characteristics. A significant longer operative time (418 vs. 337 min, p = 0.018) and less estimated blood loss (400 vs. 500 ml p = 0.038) were observed in LRC group compared with ORC group. Infection and ileus were the most common early complications after surgery. Patients underwent ORC suffered from significantly more postoperative ileus (28.0% vs. 4.8%, P = 0.038) and infection (40% vs. 9.5%, P = 0.019) than LRC group within 90 days after surgery. The mortality rate was 4.7% (1/21) and 4% (1/25) for LRC group and ORC group respectively. At a median follow-up of 21 months (range 2–61 months), the Kaplan-Meier survival curves and log-rank analysis demonstrate that there were no significant differences between the LRC and ORC groups in the 3-year overall, cancer-specific, or recurrence-free survival rates.

**Conclusions:**

It is suggested that LRC should be recommended as the primary intervention to treat muscle invasive or high risk non-muscle invasive bladder cancer in elderly patients with a relative long life expectancy.

## Introduction

With marked improvements in medical technology and health care, the average life span of the general population in most countries has progressively increased. It was reported that by 2030 in America, there will be about 72.1 million older persons, over twice their number in 2000. [1] Because of a strong link between age and bladder cancer, the incidence of bladder cancer ranked the fourth (3.69%, 1/27) and sixth (0.98%, 1/106) among all cancers in male and female over 70 years old in America, respectively. [2] Meanwhile, the incidence of bladder cancer was rising with the increasing number of older people in China, ranking 1st in the Chinese urinary malignant tumor and reaching a peak over age 85 with an incidence of 69.7/100,000 [3]. So it is important but still a conundrum for most urologists to treat elderly patients with muscle invasive or high risk non-muscle invasive bladder cancer. [4], [5].

Although open radical cystectomy (ORC) with different types of urine diversion have been proved safe for elderly patients and remains the standard of care for the treatment of muscle invasive bladder cancer, it is associated with significant short and long-term morbidity. [5], [6], [7] Elderly patients, especially those older than 75 years old, are always associated with several comorbidities, thus putting these patients at an even greater risk of complications or mortality. [8], [9] As a result, older patients may potentially be guided toward conservative therapies such as radiation therapy with or without chemotherapy, or palliative transurethral resection. [7] It is imperative for us to find ways minimizing the perioperative morbidity and mortality in elderly patients. Recently, laparoscopic surgery has been widely accepted as a minimally invasive treatment to reduce the morbidity after conventional surgery, and a number of studies have demonstrated the feasibility of laparoscopic radical cystectomy (LRC) was technically feasible and oncological safe since the first report by Parra et al. [10] in 1992 [11]–[15].

The elderly patient poses several challenges to LRC surgery such as whether elderly patients can tolerate longer operation time, pneumoperitoneum, and peculiar surgical position as well as younger patients. Few studies, however, have focused the feasibility of LRC on the elderly patients older than 75-year-old compared with ORC. To explore the morbidity, mortality and oncological results of LRC in the elderly patients more than 75-year-old, we thus conducted such a retrospective single center study with a control group of ORC elderly patients.

## Materials and Methods

Between January 2009 and December 2013, 310 consecutive patients underwent LRC or ORC and urinary diversion in our institution, the median age was 64 years old (range 31 to 89). Of these patients, 54 patients were older than 75-year-old, 24 and 30 were included in the LRC group and ORC group respectively. All the participants were informed that their clinical information may be used in later clinical study when they enter hospital and their written informed consents were obtained. This study was approved by the ethical board of Changhai Hospital, all procedures performed in accordance with the ethical principles expressed in the 1995 Declaration of Helsinki. Patients’ information was anonymized prior to analysis. The indication for radical cystectomy was histologically diagnosed muscle-invasive bladder cancer by transurethral resection, or biopsy confirmed recurrent multifocal high-grade superficial bladder cancer or bladder cancer in situ that were refractory to repeated transurethral resection. The clinical and follow-up data were retrospectively collected and analyzed from our bladder cancer database. We failed to contact 3/24 and 5/30 patients in LRC group and ORC group respectively to confirm the survival status and late complications, thus they are excluded from the study.

All patients underwent a thorough preoperative examination including routine laboratory test, chest radiography, and intravenous pyelogram, echocardiography, lung function test, computerized tomography or magnetic resonance imaging and abdominal ultrasonography. Patients were graded according to the American Society of Anesthesiologists (ASA) class. Common comorbidities such as hypertension, coronary artery disease, chronic obstructive pulmonary disease, diabetes mellitus and other chronic diseases were recorded.

Two days before surgery all patients began Semi-liquid diet and metronidazole intake. The day before surgery, patients began liquid diet and underwent bowel preparation with 50% magnesium sulfate 12 hours before surgery. All patients wore compression stockings before entering operation room and broad-spectrum systemic antibiotic was given intravenously during induction of anesthesia. The procedure of LRC and ORC with bilateral pelvic and iliac lymphadenectomy for man and woman was performed according to the procedures described by Campbell-Walsh Urology. [16] The urinary diversion of LRC was reconstructed extracorporeally through a 5 to 6 cm supraumbilical extended incision of the camera port. All the surgeons (XCL, WLH, HJG) are urological professor performing cystectomy more than 25 cases annually, two of the surgeons (XCL, WLH) in this series performed both ORC and LRC surgery, while the other one performed only open surgery (HJG).

The demographic parameters included age, gender, body mass index (BMI), comorbid conditions, surgery history, laboratory test results, multidisciplinary consultations needed. Operative variables namely operative time (defined as anesthesia from begin to end) and estimated blood loss (EBL), and transfusion. Perioperative outcome such as time to liquid intake, time to exsufflation, time to canalization and hospital stay after surgery. Early complication was defined within 90 days and late complications occurred more than 90 days after operation. [5] Oncological outcomes, including survival and recurrence were evaluated. The pathological tumor stage and grade were examined according to the TNM classification and the World Health Organization system in 2004, respectively. [17], [18].

Chi-square test, Mann-Whitney U test and Student t test were used to compare categorical, nonparametric, and parametric data respectively between the two groups. Overall, disease-specific and recurrence-free survival were analyzed using Kaplan-Meier survival method and log-rank tests. Differences were considered significant at p<0.05. Statistical analysis was performed with the use of the Statistical Package for Social Scientists, version 19.0 (IBM Inc).

## Results

As was shown in Table 1, patients in the LRC group and ORC group had comparable preoperative characteristics with regards to patients’ age, gender, BMI, ASA class, comorbid conditions, surgical history and the results of blood test. Pathological results were also comparable between the two groups as was showed in Table 2.

**Table 1:** Baseline patient characteristics.

	LRC	ORC	P value
**Age, median (IQR)**	77 (75,79)	78 (75,80)	0.750*
**Gender, men/women**	19/2	21/4	0.673**
**BMI, mean±SD (kg/m^2^)**	23.5±2.3	23.4±2.5	0.959*
**Hb, mean±SD (g/L)**	120.1±25.6	125.0±23.0	0.501*
**SCR, mean±SD(umol/L)**	96.8±62.0	90.1±31.3	0.641*
**ALB, mean±SD (g/L)**	37.1±4.2	36.8±3.2	0.756*
**MC needed before surgery (n%)**	6 (28.6%)	9 (36%)	0.592**
**ASA class (n%)**			
** 2**	10 (47.6%)	15 (60.0%)	0.401**
** 3**	11 (52.4%)	10 (40.0%)	
**Surgery history (n%)**			
** Abdominal surgery**	3 (14.3%)	5 (20.0%)	0.710**
** Nephrectomy**	1 (4.8%)	3 (12%)	0.614**
**Comorbid conditions**			
** Coronary heart disease**	2 (9.5%)	1 (4.0%)	0.585**
** Hypertension**	5 (23.8%)	11 (44%)	0.152**
** Diabetes mellitus**	7 (33.3%)	4 (16%)	0.170**
** COPD**	2 (9.5%)	1 (4.0%)	0.585**
** Other chronic diseases**	1 (4.8%)	2 (8.0%)	1.000**

LRC = laparoscopic radical cystectomy; ORC = open radical cystectomy; IQR = Interquartile range; BMI = body mass index; Hb = hemoglobin; SCR = Serum creatinine; ALB  = serum albumin; MC = multidisciplinary consultation; ASA =  American society of anesthesiologists; COPD = chronic obstructive pulmonary disease.

*Student t test was used for statistical analysis.

**Chi-square tests was used for statistical analysis.

**Table 2:** Pathological results.

	LRC	ORC	P value**
**Pathological stage (n%)**			0.883**
** T0, Ta, Tis, T1**	8 (38.1%)	12 (48.0%)	
** T2**	5 (23.8%)	6 (24.0%)	
** T3**	3 (14.3%)	3 (12.0%)	
** T4**	5 (23.8%)	4 (16.0%)	
**Grade (n%)**			1.000**
** Low grade**	0	1 (4%)	
** High grade**	21 (100%)	24 (96.0%)	
**Lymph node status(n%)**			0.585**
** negative**	19 (90.5%)	24 (96.0%)	
** positive**	2 (9.5%)	1 (4.0%)	
**Lymph node number**	12	13	0.684*
**Positive surgical margins**	0	0	1.000

*Student t test was used for statistical analysis.

**Chi-square tests was used for statistical analysis.

There was no conversion to open surgery in LRC group. The operative and postoperative characteristics were shown in Table 3. A significant difference was observed at the two groups regarding to operative time and EBL. The LRC group required a significant longer operative time, with a mean operative time of 418 min compared with 337 min for ORC group (P = 0.018). The median EBL was 400 ml for LRC and 500 ml for the ORC (P = 0.038). Compared with preoperative data, serum albumin significantly decreased 7.2±5.8 g/L and 8.7±4.4 g/L (both P<0.001) for LRC group and ORC group respectively and so was hemoglobin which significantly decreased 15.5±19.31 g/L and 27.8±19.3 g/L (both P<0.001) respectively. However, there was no significantly change for serum creatinine in both groups. Seven patients in LRC group and 16 in the ORC group required multidisciplinary consultation after operation (P = 0.038), which was resulted from more complications occurred in the ORC group.

**Table 3:** Operative and postoperative characteristics.

	LRC	ORC	P value
**Operative time, mean**±**SD (min)**	413±103.6	337±105.0	0.018*
**EBL (ml), median (IQR)**	400 (250,500)	500 (350,800)	0.038#
**Transfusion needed (n%)**	6 (28.6%)	10 (40%)	0.418**
**Division (n%)**			0.469**
** Ileal conduit**	14 (70.0%)	22 (84.6%)	
** Ureterocutaneostomy**	5 (25.0%)	3 (11.5%)	
** Orthotopic neobladder**	1 (5.0%)	1 (3.8%)	
**Hb, mean**±**SD (g/L)**	104.6±16.0	97.2±15.3	0.115*
**SCR, mean**±**SD (umol/L)**	101.7±56.8	99.8±45.7	0.900*
**ALB, mean**±**SD (g/L)**	29.9±4.2	28.0±4.0	0.133*
**MC needed after operation (n%)**	7 (33.3%)	16 (64.0%)	0.038*
**Time to liquid intake, mean**±**SD (d)**	4.5±2.5	5.1±2.1	0.361*
**Time to nasogastric tube removal,** **mean**±**SD (d)**	4.1±2.6	4.6±2.2	0.487*
**Time to canalization, mean**±**SD (d)**	11.0±4.1	11.5±6.1	0.781*
**Time to exsufflation, mean**±**SD (d)**	3.5±0.8	4.1±1.1	0.064*
**Hospital stay after surgery(d), median (IQR)**	14 (10,18)	15 (12,26)	0.232#

*Student t test was used for statistical analysis.

**Chi-square tests was used for statistical analysis.

# Mann-Whitney U was used for statistical analysis.

Postoperative complications were noted in Table 4. Infection and ileus were the most common early complications after surgery. Patients underwent ORC suffered from significantly more postoperative ileus (28.0% vs. 4.8%, P = 0.038) and infection (40% vs. 9.5%, P = 0.019) than LRC group within 90 days after surgery. In 2 LRC group and 5 ORC group patients (Table 3), a postoperative complications required re-operation. There was one patient (4.7%) in LRC group died from operation related complications. An 84 years old man died of multiple organ dysfunction caused by ileus 5 months after discharging from hospital. One (4%) 77-year-old male patient in the ORC group died of sepsis resulted from ileus within 3 months after operation.

**Table 4:** Postoperative complications.

	LRC	ORC	P value**
**Early complications 90≤ days (n%)**			
** Infection** †	2 (9.6%)	10 (40.0%)	0.019
** Ileus** ‡	1 (4.8%)	7 (28.0%)	0.038
** Anastomotic leak**	1 (4.8%)^a^	2 (8.0%)^b^	1.000
** Delirium**	1 (4.8%)	3 (12.0%)	0.614
** Arrhythmia**	2 (9.6%)	2 (8.0%)	1.000
** wound dehiscence**	0	3 (12.0%)^c^	0.239
** Hoarseness**	1 (4.8%)	0	0.457
** Diarrhea**	1 (4.8%)	0	0.457
**Late complications >90 days (n%)**			
** Pyelonephritis**	2 (9.6%)	4 (16.0%)	0.673
** Ileus**	1 (4.8%)	3 (12.0%)	0.614
** Ureteral stricture**	1 (4.8%)^d^	1 (4.0%)^d^	1.000
** Incisional Hernia**	0	1 (4.0%)	1.000
**Re-operation required**	2 (9.6%)	5 (20.0%)	0.436
**Mortality (n%)**			
** ≤90 days**	0	1 (4.0%)	1.000
** >90 days**	1 (4.8%)	0	1.000

† Patients was diagnosed as infection when they presented continuous fever and antibiotics were considered as the most effective treatment to cure it.

‡ Ileus was diagnosed when gas- fluid levels were seen in abdominal X-rays.

a Treated by fistula resection and Intestinal anastomosis.

b One of them treated by fistula resection and Ileostomy, the other one with urine leakage from ileo-ureteral anastomosis healed spontaneously.

c All Three treated with primary suture after wound debridement.

d Both of them treated by retrograde placement of single J stent with flexible cystoscopy remained indwelling for 8 weeks.

**Chi-square tests was used for statistical analysis.

**Figure 1:**
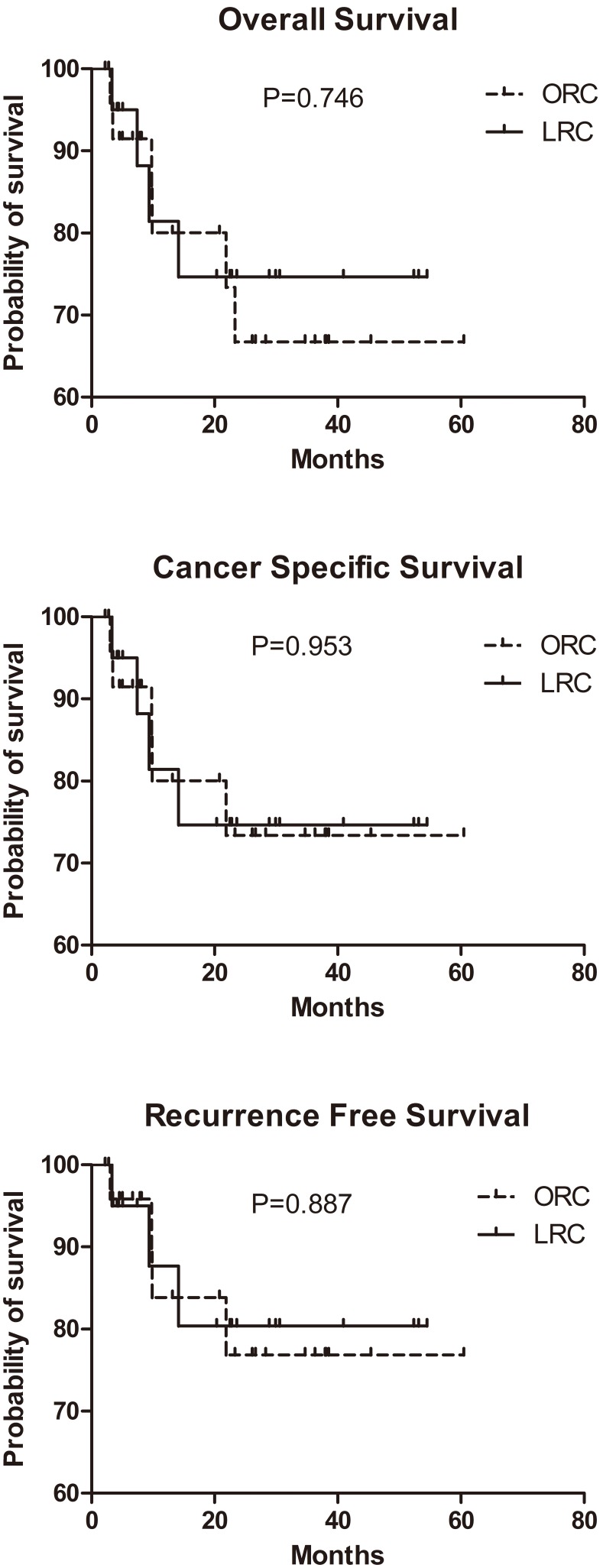
Kaplan-Meier estimate of 3-year survival for overall, cancer-specific, recurrence-free survival. Log-rank test indicates that overall survival, cancer specific survival and recurrence free survival among LRC and ORC groups are not significantly different.

The median follow-up was 20 months (range 2–55 months) and 21 months (range 3–61 months) for LRC group and ORC group respectively. There were 17 (81.0%) and 19 (76.0%) patients alive for LRC group and ORC group at the last follow-up, respectively. All patients in LRC group and the majority of patients (94.7%, 18/19) are disease-free. Cancer specific death occurred in 3/4 (75.0%) and 5/6 (83.3%) in LRC group and ORC group, respectively. Kaplan-Meier survival curves, as was shown in Figure 1, demonstrate that there were no significant differences between the LRC and ORC groups in the 3-year overall, cancer-specific, or recurrence-free survival rates.

## Discussion

In most countries life expectancy of the general population is increasing, however, it was estimated that nearly 1 in 3 people older than 70-year-old may suffer from a wide variety of malignant tumors, including bladder cancer, which were the main factor affecting the health of elderly people. [2] The incidence of bladder cancer increases distinctly with increasing age, especially those who beyond 70-year-old. [5] Elderly patients with muscle invasive or high risk non-muscle invasive bladder cancer who require major surgery such as cystectomy pose difficult challenges to the operative surgeon. This dilemma is due to the complexity of comorbidities of the elderly patients and great risk of complications and mortality in this subset of patients. [5], [9], [19] Collectively these considerations have led to delay cystectomy for elderly patients. Prout et al. [20] reported only 25% of patients with muscle-invasive bladder cancer between 70 to 79 years old were treated with radical cystectomy compared with 55% of those aged 55 to 59 years. Recently, several studies have proved that age is not a limiting factor in selecting radical cystectomy as a treatment method for muscle-invasive bladder cancer. [4], [20]–[23].

Although studies have proved that elderly patients can safely undergo cystectomy, this surgical procedure always associated with high incidence of postoperative complications and morbidity. Patients older than 75-year-old who underwent ORC suffers from 38.6% to 64% early complications and 16.4% to 42% late complications, and a mortality rate ranging from 2% to 6% within 30 days after cystectomy. [22], [24]–[27] Minimally invasive treatments such as laparoscopic surgery has been expanding rapidly, the excellent perioperative and oncological results in the treatment of renal cell cancer and prostate cancer and technical developments have paved the way to LRC. [9], [13] It has been reported that LRC could reduce blood loss, analgesic consumption, postoperative complication and promote earlier recovery of bowel function and return to normal activity [9], [13], [28]. Despite these advantages, ORC has still been considered to be the standard care for muscle-invasive or high-risk non muscle-invasive bladder cancer, especially for elderly patients. [6] Several concerns contribute to this situation such as whether elderly patients can tolerate longer anesthetic period and the steep Trendelenburg position, and whether the older patients with limited renal function reserve can tolerate metabolic acidosis caused by pneumoperitoneum. [9] However, as far as we know, comparative data in terms of morbidity rate and long term survival benefit for LRC and ORC procedures in patients aged older than 75 years are presently lacking.

To the best of our knowledge, we found Richards et al. [9] conducted the first observation to justify the feasibility of robot-assisted radical cystectomy (RRC) compared with ORC in elderly bladder cancer patients. In spite of longer operative time, it was reported that RRC could achieve similar perioperative outcomes without compromising pathologic outcomes, nevertheless, with less blood loss (275 vs. 600 ml), shorter hospital stays (7 vs. 14.5 days) and less major complications (10% vs. 35%) compared with ORC. Richards et al. [9] speculated that RRC was associated with fewer complications than ORC in the elderly was related to less fluid shifts, decreased operative blood loss, and lower rate of transfusions. Although RRC is able to bridge the technical difficulties of conventional LRC, and facilitate a broader diffusion of minimally invasive treatment for muscle-invasive bladder cancer, it is still not available worldwide. In the present study, it was observed that LRC group associated with less EBL, and could significantly reduce the incidence of ileus and perioperative infection within 90 days after surgery in contrast to ORC group. What is more, patients in LRC group recovered more smoothly than ORC group, as was indicated by less multidisciplinary consultation needed after operation (33.3% vs. 64.0%, P = 0.038). Interestingly, it was found that serum albumin both reduced 7.2 to 8.7 g/L in LRC and ORC group respectively. It was thought that serum albumin reflects nutritional status, and strongly associated with 90-day mortality, accounting for a 2.5-fold increase in the risk of 90-day mortality per7 g/L decrease in serum albumin. [19] So it is necessary that attention should be paid to keep a balanced nutrition after surgery in order to reduce the possibility of delayed recovery.

Despite less complication for LRC group, the hospital stay was similar to ORC group in this study. This was mainly due to the different health-care system compared with the United States or European practice. The mean hospital stay after surgery in our institution was 14 and 15 for LRC and ORC group respectively, which was obviously longer than previous report. [9], [13], [23], [28], [29] The healthcare system is different between China and US, patients in our institution often comes from different provinces, it’s not convenient for patients to find an rehabilitation center for medical support after discharging from hospital, so most patients preferred to stay until all the tubes and sutures were removed even if they were fit for discharge in the LRC group. Hemal et al. [30] and Saika et al. [31] considered hospital stay was sometimes a reflection of health-care mode and the prevalent social condition of the patients. Overall survival rate is the golden standard to evaluate the feasibility of a medical intervention, it was observed that the LRC not only achieved less complication but also gained comparable overall survival (81% vs. 76%, P = 0.746) compared with ORC at a median follow-up of 21 months (range 3–61 months).

The limitations of this study should be noted. First of all, the nature of a retrospective study made it impossible to avoid the selection bias and attrition bias. A randomized, prospective study with age, medical comorbidity and urinary diversion stratification, and longtime follow-up would be better to assess the effect of LRC for elderly patients. Secondly, the sample size of this study was small. In most cases, patients more 75-year-old, especially those with worse physical condition, were reluctant to undergo such a major surgery. So the results maybe only reflect the outcome of those elderly patients with better physical condition and more optimistic attitude. Thirdly, although intensive care unit (ICU) monitoring period was a reliable indicator to evaluate the condition of a patient after surgery, all patients underwent cystectomy in our institution routinely stayed in ICU for the first day after surgery. Only if patient underwent a re-operation, he would be sent to ICU again. So the clinical practice of our institution made it less valuable to include this parameter.

## Conclusions

The results of this study displays the oncological efficacy of LRC is similar to that of ORC, while with less postoperative complications and blood loss. We suggest that it is promising to accept LRC as the primary intervention to treat muscle invasive or high risk non-muscle invasive bladder cancer in elderly patients with a relative long life expectancy and multidisciplinary cooperation was required to perform a cystectomy for an elderly patient. However, larger sample size and prospective randomized studies are needed to confirm these results.
